# When Water Availability Is Low, Two Mediterranean *Salvia* Species Rely on Root Hydraulics

**DOI:** 10.3390/plants10091888

**Published:** 2021-09-13

**Authors:** Elisa Abate, Maria Azzarà, Patrizia Trifilò

**Affiliations:** Dipartimento di Scienze Chimiche, Biologiche, Farmaceutiche ed Ambientali, Università di Messina, Viale Ferdinando Stagno d’Alcontres 31, 98166 Messina, Italy; elisa.abate@unime.it (E.A.); mariaazzara960612@gmail.com (M.A.)

**Keywords:** climate change, Mediterranean species, membrane damages, plant hydraulic conductance, rehydration capacity, water content

## Abstract

Increase in severity and frequency of drought events is altering plant community composition, exposing biomes to a higher risk of biodiversity losses. This is exacerbated in the most fragile areas as Mediterranean biome. Thus, identifying plant traits for forecasting species with a high risk of drought-driven mortality is particularly urgent. In the present study, we investigated the drought resistance strategy of two Mediterranean native species: *Salvia ceratophylloides* Ard. (*Sc*) and *Salvia officinalis* L. (*So*) by considering the impact of drought-driven water content decline on plant hydraulics. Well-watered samples of *Sc* displayed higher leaf and stemsaturated water content and lower shoot biomass than *So* samples, but similar root biomass. In response to drought, *Sc* showed a conservative water use strategy, as the prompt stomatal closure and leaves shedding suggested. A drought-tolerant mechanism was confirmed in *So* samples. Nevertheless, *Sc* and *So* showed similar drought-driven plant hydraulic conductance (K_plant_) recover ability. Root hydraulic traits played a key role to reach this goal. Relative water content as well as loss of cell rehydration capability and membrane damages, especially of stem and root, were good proxies of drought-driven K_plant_ decline.

## 1. Introduction

In the last decades, the increase in severity and frequency of drought events is exposing vegetation to a higher risk of drought-driven die-off [1,2,3]. According to climate projections, global warming is not expected to be homogeneous: higher increase in temperature and drought events have been forecasted for the Mediterranean region e.g., [4,5,6]. This, in turn, may lead to more relevant negative feedback on biodiversity richness of the Mediterranean biome, exacerbating the recorded ongoing vegetation pattern shifts [7,8,9,10] and increasing the extinction risk of endemic flora [11,12,13]. Mediterranean region shows high levels of plant diversity and endemism, as a result of different co-occurring factors [14,15]. The high numbers of endemic plant species are adapted to cope with warm and frequently long dry periods that typically occur in the Mediterranean biome. Thus, the Mediterranean biodiversity hotspot is coupled to different specific adaptive strategies for delaying and/or tolerating tissue dehydration i.e., [16,17,18,19,20,21]. However, increasing temperature and drought events may lead to exceeding the species-specific drought resistance threshold. Vegetation responses to ongoing climate change is a complex process involving the coordination of different and not well-understood physiological mechanisms. Plant hydraulics play a critical role in vegetation ability to cope with drought [22] and hydraulic failure is considered the major driver of vegetation die-off [23,24]. Nevertheless, many questions on plant hydraulics remain unresolved [22,25] and looking for a robust proxy for predicting plant die-back is urgent.

Changes in plant water content in response to drought have recently received a renewed attention [26]. Plant water status is linked to different key physiological mechanisms, including water transport and its regulation as well as carbon metabolism [27,28]. On this view, the relative water content (RWC) has been suggested as a simple indicator of plant mortality risk. However, the species-specific cell desiccation tolerance is still largely unknown. To the best of our knowledge, only few studies have investigated water content thresholds leading drought-induced mortality risk and it is unclear if a specific organ (leaf, stem, root) or whole plant water content can be an actual proxy of mortality risk. Rosner et al. [29] reported good correlations between stem RWC and the loss of hydraulic conductivity in certain tree species. However, in a most recent study, Mantova et al. [30] indicated that stem RWC is useful for predicting the loss of hydraulic conductivity in woody angiosperms but not in conifers.

In the present study, we reported the hydraulic performance, including changes in relative water content, of two native Mediterranean species, *Salvia ceratophylloides* Ard. (*Sc*), a perennial herbaceous species [31,32,33], and *Salvia officinalis* L. (*So*), a perennial evergreen subshrub [34], experiencing mild and severe drought events and then rewatering. In detail, we compared the drought resistance strategy of *Sc* versus *So* (i.e., a drought-tolerant species [35,36]) and the plant recovery ability of the two *Salvia* species in order to investigate if and how mild or severe leaf hydraulic impairment and/or loss in cell rehydration ability can affect the whole plant hydraulics. Similar whole-plant drought vulnerability is expected on species with similar leaf hydraulic safety [37,38,39]. However, to the best of our knowledge, no study has focused on—species showing a moderate succulence syndrome. Recently, Abate et al. reported higher leaf succulence in *Sc* versus *So* [40]. This, in turn, led to different RWC thresholds but similar leaf water potential and leaf water content for leaf dehydration vulnerability in the two *Salvia* species [40]. On this basis, we tested the impact of leaf hydraulic impairment on the whole plant drought vulnerability in *Sc* and *So*. Moreover, we checked: (i) possible relations between leaf, stem and root drought-driven water content and/or loss in cell rehydration ability and plant hydraulics of the two *Salvia* species in order to test possible tool(s) for monitoring the plant die-back and, then, for predicting the drought-driven risk of mortality; (ii) if hydraulic traits and/or drought resistance mechanism of *Sc* may expose this species to potentially higher risk of extinction under predicted climate change scenario. *S. ceratophylloides* is a rare endemic perennial herbaceous species of southern Italy [31]. Until 2008, such a species had been considered “extinct in the wild” but most recent studies documented the presence of natural populations in its native area (i.e., Calabria, Italy), suggesting that the vulnerability of *S. cerathophylloides* has been likely induced by anthropogenic causes (as an improper use of the soil) more than an unsuited species-specific water use strategy [32,33].

## 2. Results

*S. ceratophylloides* and *S. officinalis* differed strongly in biomass and structural traits (Table 1). *So* showed higher shoot biomass compared to *Sc*. Significantly higher values of number of leaves per plant (about 165 versus 50), whole plant leaf area (2800 versus 800 cm^2^), leaves dry weight (13 versus 2 g), and stem dry weight (1.1 versus 0.3 g) were recorded in *So* versus *Sc*. Moreover, a different shoot biomass allocation was recorded: *Sc* showed a statistically significant two-fold higher stem/leaf ratio than *So* (Table 1). The two study species showed similar root dry mass (i.e., about 1.3 g) but a 3-times higher root/shoot ratio value was recorded in *Sc* compared to *So* plants. *Sc* showed a higher leaf and stem-saturated water content (SWC) values than *So*. By contrast, not statistically significant different values of root SWC were recorded in the two species.

**Figure 1:**
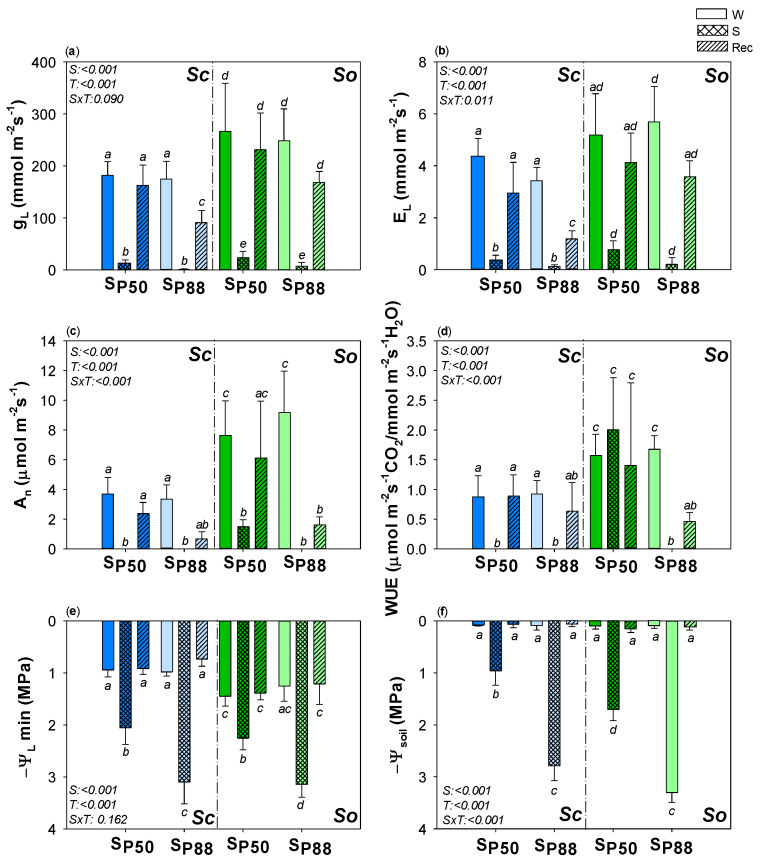
Effect of experimental treatments on gas exchange and water potential. Mean ± SD (*n* = 6) values of: (**a**) midday leaf conductance to water vapor, g_L_; (**b**) transpiration rate, E_L_, (**c**) photosynthetic rate, A_n_, (**d**) water use efficiency, WUE, (**e**) leaf water potential, Ψ_L_ and (**f**) soil water potential, Ψ_soil_ as recorded in well-watered (W, none pattern), water–stressed (S, slanting dash) and re-irrigated (Rec, mesh dash) plants of *S. ceratophylloides* (*Sc*, blue columns) and *S. officinalis* (*So*, green columns) submitted to two different water stress levels, i.e., S_P50_ and S_P80_ (for details, see the text). *p* values as obtained by the two-way ANOVA analysis are reported. Different letters indicate statistically significant differences between groups.

The higher biomass values recorded in *So* versus *Sc* was likely due to the result of higher photosynthesis rate (A_n_) and water use efficiency (WUE) values as recorded in well-watered samples of *So* versus *Sc* (Figure 1). In response to mild (S_P50_) and severe (S_P88_) drought events, strong decreases in stomatal conductance to water vapor (g_L_), transpiration rate (E_L_) and photosynthetic rate (A_n_) were recorded in the two study species. However, in response to mild water stress, higher values of gas exchange and WUE occurred in *So* versus *Sc*. Moreover, a different drought sensitivity of the two *Salvia* species was recorded. In response to water shortage, a prompt stomatal closure occurred in *Sc* but not in *So* (Figure S1). As a consequence, at S_P50_ (i.e., Ψ_L_~−2 MPa), *So* showed g_L_ loss of about 70%, while in *Sc* samples, cuticular conductance values were already recorded. S_P50_ treatment did not induce permanent damages in the two *Salvia* species. After a week of re-irrigation, S_P50_ samples showed all measured parameters statistically similar to values recorded before drought. By contrast, no full recovery of gas exchange and WUE was recorded after the applied severe water stress, despite the full recovery of the leaf water potential. 

**Figure 2:**
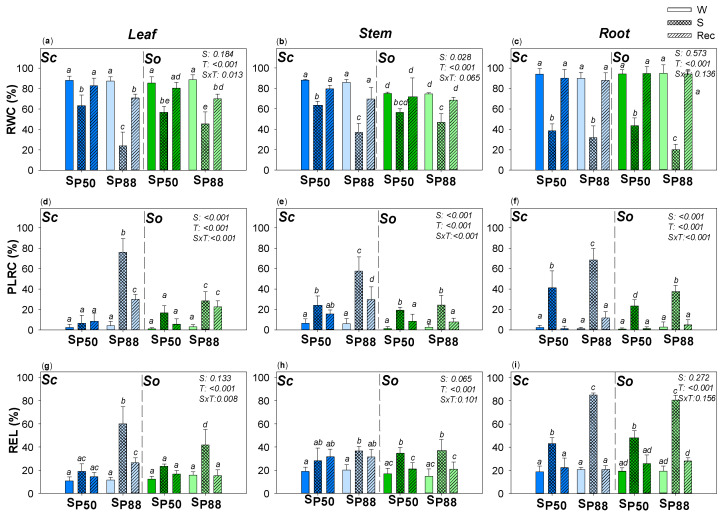
Effect of experimental treatments on water content and drought-driven damages. Mean ± SD (*n* = 5) values of relative water content (RWC); percentage loss of rehydration capability (PLRC) and relative electrolyte leakage (REL) as recorded in leaf (**a**,**d**,**g**), stem (**b**,**e**,**h**) and root (**c**,**f**,**i**) samples of well-watered (W, none pattern), water-stressed (S, slanting dash) and re-irrigated (Rec, mesh dash) plants of *S. ceratophylloides* (*Sc*, blue columns) and *S. officinalis* (*So,* green columns) submitted to two different water stress levels, i.e., S_P50_ and S_P80_ (for details, see the text). *p* values as obtained by the two-way ANOVA analysis are reported. Different letters indicate statistically significant differences between groups.

Applied drought treatments strongly affected the relative water content of leaf, stem and root samples of the two *Salvia* species (Figure 2). However, after the rewatering, a full recovery of root RWC values was recorded, including when a severe water stress was experimented by *Sc* and *So* samples. This recovery was coupled to new roots growth, as observed in both species (data not shown). A different behavior was observed in the stem and leaf samples of the two *Salvia* species, especially in response to severe drought treatment. Water stress inducing 50% loss of leaf hydraulic conductance (K_L_, i.e., S_P50_) did not affect the leaf and stem cell ability to recover water content in *Sc* and *So* samples and no permanent damage was recorded after rewatering in the two *Salvia* species. By contrast, experiencing about 88% loss of leaf hydraulic conductance (i.e., S_P88_), only a partial recovery of leaf RWC values in *Sc* as well as *So* samples was recorded. The inability to recover the leaf water content was likely induced by a residual 20% loss in cell rehydration capability, as recorded in the two species even after rewatering. However, leaf *Sc* samples were more severely affected by S_P88_ treatment. In accordance, S_P88_ leaf samples of *Sc* showed higher leaf cell membrane damages (i.e., REL ~ 70% versus 40%, respectively) and higher percentage loss of cell rehydration capability (PLRC) values (i.e., 80% versus 20%, respectively) compared to *So* S_P88_ leaf samples. Moreover, *So* stem samples were not permanently affected by experiencing severe water stress. By contrast, in Rec_SP88_ *Sc* stem samples, a residual 20% PLRC and REL values as high as about 35% were recorded.

**Figure 3:**
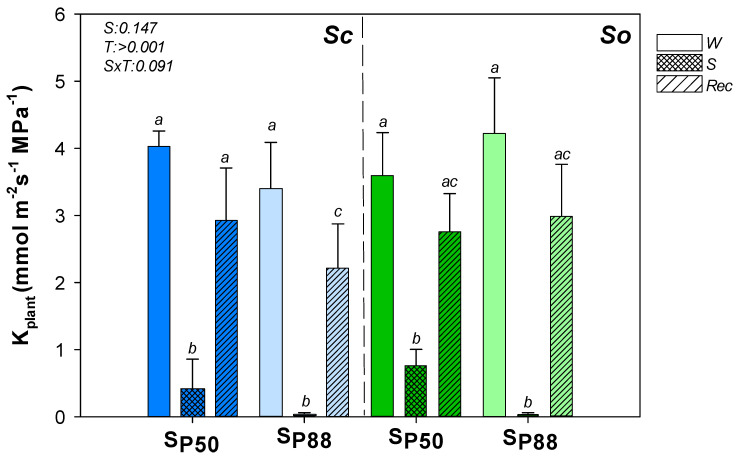
Effect of experimental treatments on plant hydraulic conductance. Mean ± SD (*n* = 6) values of plant hydraulic conductance (K_plant_) as recorded in well-watered (W, none pattern), water–stressed (S, slanting dash) and re-irrigated (Rec, mesh dash) plants of *S. ceratophylloides* (*Sc*, blue columns) and *S. officinalis* (*So*, green columns) submitted to two different water stress levels, i.e., S_P50_ and S_P80_ (for details, see the text). *p* values as obtained by the two-way ANOVA analysis are reported. Different letters indicate statistically significant differences between groups.

*So* and *Sc* well-watered samples showed similar plant hydraulic conductance (K_plant_) values (Figure 3). Moreover, similar K_plant_ values were recorded when the two *Salvia* species were submitted to the two drought treatments and then re-irrigated (Figure 3).

**Figure 4:**
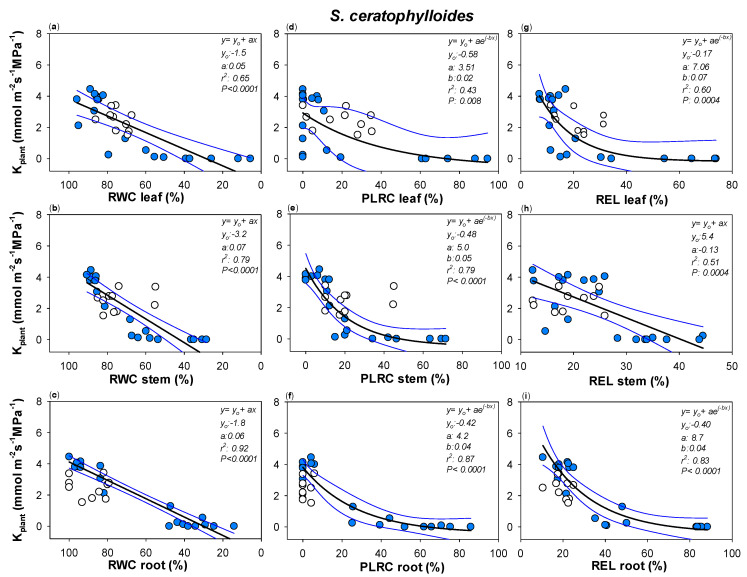
Relationships between plant hydraulic conductance (K_plant_) and (**a**) leaf, (**b**) stem and (**c**) root relative water content (RWC leaf, RWC stem and RWC root, respectively), (**d**) leaf, (**e**) stem and (**f**) root percentage loss of leaf rehydration capacity (PLRC leaf, PLRC stem, PLRC root) and (**g**) leaf, (**h**) stem and (**i**) root relative electrolyte leakage (REL leaf, REL stem and REL root) as measured in *S. ceratophylloides*. Best fitted regression curves are designed by dark line. Confidence intervals are designed by blue lines. White symbols indicate data recorded in re-irrigated samples and not included in the regression. Regression equation, coefficient values, correlation coefficients (r^2^) and *p* values are also reported.

**Figure 5:**
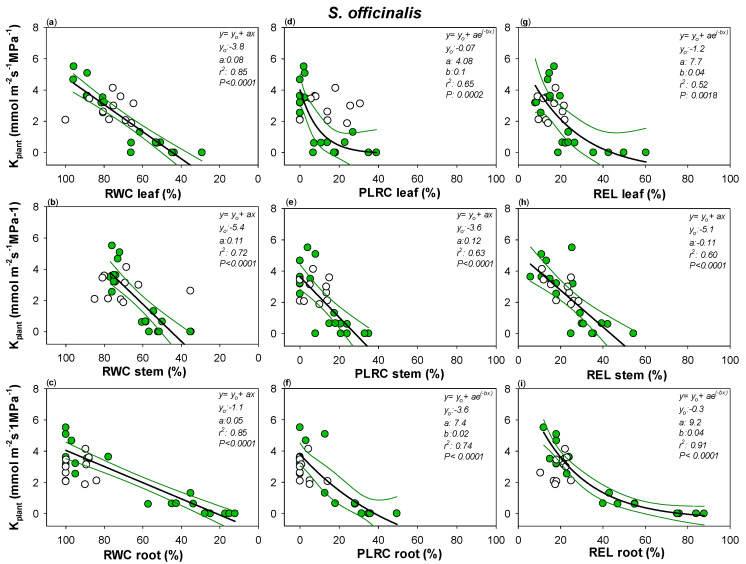
Relationships between plant hydraulic conductance (K_plant_) and (**a**) leaf, (**b**) stem and (**c**) root relative water content (RWC leaf, RWC stem and RWC root, respectively), (**d**) leaf, (**e**) stem and (**f**) root percentage loss of leaf rehydration capacity (PLRC leaf, PLRC stem, PLRC root) and (**g**) leaf, (**h**) stem and (**i**) root relative electrolyte leakage (REL leaf, REL stem and REL root) as measured in *S. officinalis*. Best fitted regression curves are designed by dark line. Confidence intervals are designed by green lines. White symbols indicate data recorded in re-irrigated samples and not included in the regression. Regression equation, coefficient values, correlation coefficients (r^2^) and *p* values are also reported.

Robust correlations were recorded between K_plant_ and leaf, stem and root relative water content in the two *Salvia* species (Figure 4 and Figure 5). Drought-driven K_plant_ decline was clearly strongly related to the cell loss rehydration capability as well as to drought-driven cell membrane damages. RWC, PLRC and REL thresholds of K_plant_ impairment were similar among the three plant organs as well as between *Sc* and *So* (Figure 4 and Figure 5, Table S1). Only leaf RWC value leading to 80% loss of K_plant_ was significantly lower in *Sc* versus *So*, as well as no confidence intervals overlapping being recorded between *Sc* leaf versus *So* root REL value leading to 50% loss of K_plant_ (Figure 4 and Figure 5, Table S1). Nevertheless, overall, in all three plant organs, RWC values as low as about 65% as well as PLRC values of about 15% led to K_plant_ loss of 50% in the two *Salvia* species.

It can be noted that large confidence intervals were recorded between leaf PLRC and REL values and the corresponding plant hydraulic conductance declines. By contrast, most robust correlations were recorded between K_plant_ and stem and root samples.

## 3. Discussion

**Figure 6:**
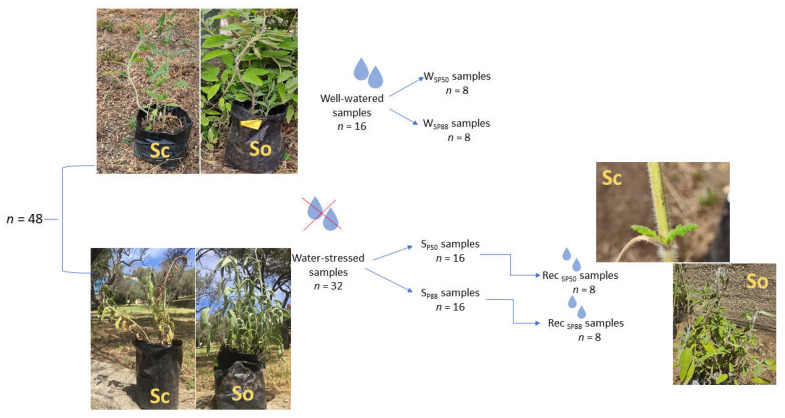
Experimental design: 48 potted samples of *S. ceratophylloides* (*Sc*) and *S. officinalis* (*So*) were divided into two groups: watered samples (*n* = 16) and water–stressed samples (*n* = 32). Watered samples (W) were regularly irrigated at field capacity during the experimental period and measured, as control samples, at the same time as water–stressed samples (i.e., 8 samples for each drought treatment). Water–stressed samples were further divided into two groups subjected to a mild (i.e., S_P50_ samples, *n* = 16) and a severe (S_P88_, *n* = 16) water-stress treatment, respectively. A subset of these samples (*n* = 8) was measured when the imposed water stress level was reached, while the other subset of drought-treated samples (*n* = 8) was re-irrigated at field capacity (i.e., Rec _SP50_ and Rec _SP88_ samples) and then measured.

*S. ceratophylloides* and *S. officinalis* exhibited different resistance mechanisms for coping with drought but a similar plant hydraulics recovery ability, especially in response to severe drought. In detail, recovery from mild water stress led to similar water content values in the three plant organs of the two *Salvia* species. However, different gas exchange and water use efficiency values occurred in *Sc* versus *So*. By contrast, experiencing about 80% of K_L_ loss caused different residual leaf and stem cell damages in *Sc* versus *So* samples but similar impact on gas exchange. Nevertheless, a similar K_plant_ recovery was recorded in *Sc* and *So* in response to the two drought recovery treatments and no plant death was recorded after a month by the end of the experimental period (personal observation). Hydraulic recovery was obtained by new roots production and, then, renewed root functioning in *Sc* and *So* samples. This avoided permanent loss in root cell rehydration capability and cell membrane damages in fine roots (i.e., site of water and nutrient uptake). Overall, these results strongly suggest that root hydraulics plays a key role in whole-plant recovery ability of the two Mediterranean native *Salvia* species. Moreover, our findings highlight that drought-driven changes in leaf RWC and PLRC values do not always provide proxy of plant hydraulic failure, especially when succulent and/or water saving species are considered. In accordance, likely as a consequence of relevant cell damages, a consistent leaf shedding in *S. ceratophylloides* S_P88_ samples occurred (Figure 6). Nevertheless, after a week of rewatering, new sprouting leaves were observed in *Sc* Rec_SP88_ samples (Figure 6). On this view, leaf hydraulic impairment weakly affected plant hydraulic conductance decline in *Sc* (i.e., a water saver species showing a moderate succulence syndrome). Drought resistance strategy shown by *Sc* may affect its survival under frequent and extreme drought events.

### 3.1. Two Different Drought Resistance Strategies but a Similar Root Hydraulics Recovery Ability

*S. ceratophylloides* invested less in biomass accumulation than *S. officinalis*, as lower LDMC, SDMC and RDMC values suggested. This was likely the result of lower stomatal conductance, photosynthesis rate and WUE as recorded in well-watered *Sc* versus *So* samples. These findings, along with the different leaf and stem SWC values recorded in the two *Salvia* species, confirmed a more pronounced succulent syndrome in *S. ceratophylloides* than in *S. officinalis* [40]. Overall, our results lead to consider *S. ceratophylloides* a resource-conserving species. By contrast, *S. officinalis* exhibited a resource acquisitive strategy [41]. Well-watered samples of *So* showed higher A_n_, WUE as well as leaf, stem and root dry matter content values than *Sc* plants. Moreover, *So* showed a lower stomatal conductance reduction in response to mild drought: this led to higher gas exchange than *Sc*. Thus, our data confirmed a drought-tolerant mechanism in *So* samples [35,36]. By contrast, a water-saving strategy was recorded in *Sc*. In response to mild stress, full stomatal closure was recorded in this species, and in response to severe stress, leaves shedding occurred. To avoid losses in carbon gain, species adapted to water shortage can increase their water-use efficiency (WUE) i.e., [42]. This strategy can be coupled to specific anatomical traits (as high vessel density and fibers and then high leaf mass per area) aimed at minimizing water loss i.e., [43]. In accordance, leaf mass area (LMA) increases along aridity gradients at a global scale [41]. However, *Sc* showed a lower LMA value than *So* [40] as well as a relevant reduction in WUE values in response to mild drought. Thus, the strategy adopted by *Sc* to cope with drought may explain the limited diffusion of this species in the Mediterranean region. The prompt stomatal closure, typically recorded in the water-saving species, as well as the inability to improve WUE under mild stress led to an unavoidable decrease in carbon uptake. In the long term, especially after several severe drought events, this reduction in carbon assimilation may limit the sustainability of plant metabolism and its ability to recover from drought. This, in turn, may increase the chance of plant die-back. On this view, water-saving strategy is actually less efficient than drought-tolerant mechanisms [44].

Nevertheless, similar plant hydraulic conductance declined, and mainly, similar hydraulic recovery ability in response to drought-re-irrigation treatment was recorded in the two *Salvia* species. In response to a mild drought, no permanent damages of K_plant_ were recorded. Moreover, drought-driven relevant leaf hydraulic dysfunction (i.e., K_L_ loss ~88/%) led to similar residual K_plant_ loss of about 40% in *Sc* and *So*. The recovery of the two *Salvia* species was obtained mainly by root hydraulics recovery. *Sc* and *So* root hydraulics did not remain negatively affected, including by a severe drought event. Root hydraulic conductance recovery can be obtained with the growth of new roots thus by-passing the irreversible and permanent drought-driven damages occurring at root level [45,46,47,48,49]. However, root hydraulics can be restored before growing new roots by renewing the permeability of damaged roots, as documented in succulent *Agave* and *Opuntia* [45,49]. Our results strongly confirm that a high root biomass allocation, as recorded in both species, plays a key role in coping drought [50,51,52], and highlight the urgency to fill gaps in our knowledge on the relevance of root hydraulics in regulating whole plant hydraulics, especially under drought [47,53,54,55,56]. Additional experiments aimed to investigate coupled physiological and morphological root traits, and especially their changes in response to drought, are needed to improve our lacking knowledge on this topic.

### 3.2. Water Content and Loss in Rehydration Capability Actually Drive Plant Hydraulics

Water content, but also loss of rehydration capability and cell membrane damages, were actually a proxy of the drought-driven plant hydraulic conductance decline in the two *Salvia* species. However, despite robust correlations occurring in all three organs, higher correlation values were recorded in root samples of the two *Salvia* species. Moreover, the recorded large confidence intervals in the relationships between the drought-driven leaf PLRC and REL increases and the corresponding K_plant_ declines suggested a low reliability of these leaf parameters as indicator of plant hydraulic failure. Conversely, in water saving species, such as *Sc*, leaf shedding occurs promptly to reduce water loss, and at the same time, can limit xylem embolism spread. This, in turn, avoids that hydraulic failure extends to the more carbon-expensive organs, according to the “hydraulic segmentation hypothesis” [57]. As a consequence, in this case, the leaf hydraulic failure may not necessarily lead to unavoidable plant hydraulic failure, but it may be the signal of the implementation of an adaptive strategy aimed to maintain adequate plant water content and/or tolerate substantial water losses and tissue dehydration. On this basis, this specie-specific drought-resistance strategy may overshadow link(s) between drought-driven plant decline and leaf hydraulics traits.

## 4. Materials and Methods

### 4.1. Plant Material and Growth Conditions

Experiments have been performed on 48 samples per species of *S. ceratophylloides* (*Sc*) and *S. officinalis* (*So*) plants. Seeds were planted in greenhouse trays in October 2019, after maintaining them immersed in water for 24 h. A month from sprouting, each seedling was transferred to a 3.4 L-pot, filled with forest soil collected from Colli San Rizzo (Messina, Italy) and grown in a greenhouse until the beginning of May 2020. The greenhouse received natural light, with maximum daily values of photosynthetic photon flux density (PPFD) averaging 810 ± 260 μmol s^−1^ m^−2^, air temperature ranging from 21 ± 2 °C to 17 ± 2 °C (day/night), and air relative humidity of 55 ± 3%.

In May 2020, the samples were transferred to a garden of the Department CHIBIOFARAM, University of Messina, Italy, and regularly irrigated at field capacity for a month. Then, in June 2020, *Sc* and *So* plants were randomly divided into two groups (Figure 6). One group (*n* = 16) was regularly irrigated at field capacity during the entire experimental period (i.e., watered samples, W). The second group (i.e., water-stressed samples, S, *n* = 32) was further divided into two groups (*n* = 16) submitted to two different levels of water stress (Figure 6). Specifically, water stress was induced by withdrawing irrigation until the two *Salvia* species reached the leaf water potential (Ψ_L_) inducing about 50% (i.e., Ψ_L_~−2.0 MPa, S_P50_ samples) and 88% (i.e., Ψ_L_~−3.1 MPa, S_P88_ samples) loss of leaf hydraulic conductance, K_L_, as recorded by some of us in a precedent study [40]. Then, a subset of S_P50_ (*n* = 8) and S_P88_ samples (*n* = 8) was measured (see below) and the other subset of S_P50_ (*n* = 8) and S_P88_ samples (*n* = 8), was re-irrigated and measured after 7 days (i.e., Rec_SP50_ and Rec_SP88_ samples, respectively).

During the experimental period, no rainy events occurred. The temperature ranged from 18 to 26 °C and the mean relative humidity was 65 ± 3.2% (weather station of Torre Faro, Messina, Italy).

### 4.2. Gas Exchange and Water Status

Preliminary measurements on plants of similar age and growth conditions of those used in this study showed that S_P50_ value was reached after 5–6 days and 3–4 days from withholding water in *Sc* and *So*, respectively, while S_P88_ value was recorded after 10–11 days in *Sc* and after 7–8 days in *So* from suspending irrigation. To avoid defoliation, we monitored the leaf water potential (i.e., Ψ_L_) declines on two leaves per day, as collected from different plants, starting from the 4th and 2nd day from withholding irrigation in S_P50_ *Sc* and *So* samples, respectively. Similarly, Ψ_L_ value of S_P88_ samples were measured starting from the 8th and 5th day from suspending irrigation in *Sc* and *So*, respectively. This experimental procedure led us to remove no more than 2 leaves per sample.

Leaf water potential was measured by a portable pressure chamber (3005 Plant Water Status Console, Soilmoisture Equipment Corp., Goleta, CA, USA).

Ψ_L_ as well as leaf conductance to water vapor (g_L_), transpiration rate (E_L_) and photosynthetic rate (A_n_) were measured at midday in W, S and re-irrigated samples using a portable LCi Analyzer System (ADC Bioscientific Ltd., Herts, UK). The water use efficiency (WUE) of each measured plant was estimated by the ratio: A_n_/E_L_. At least six plants per species and per treatment were measured.

### 4.3. Estimating the Relative Water Content, Rehydration Capacity and Cell Membrane Integrity of Leaf, Stem and Root Samples Experiencing Drought-Recovery Treatment

Immediately after gas exchange and water status measurements and on the same W, S and Rec measured samples, the soil was gently removed from the root system and at least 2 samples of about 2 cm-long root, stem samples, and 2 leaves per plant and treatment were collected for RWC, PLRC and REL measurements.

RWC was calculated as: 100 × [(FW − DW)/DW]/SWC and PLRC as: 100 × 100 − [(TW − DW)/DW]/SWC where FW is the fresh weight (i.e., the sample weight as measured immediately after sampling), TW is the turgid weight (i.e., the sample weight as measured after maintaining the petiole or the whole stem and root sample immersed in deionized water for at least 8 h), DW is the dry weight (i.e., the oven-dried sample weight) and SWC is the saturated water content (i.e., TW/DW, g g^−1^) of sample at full turgor. Applied formula for estimating RWC allowed us to avoid mistakes as caused by cell loss rehydration ability, especially in low water status samples [40]. Cell membrane integrity was indirectly estimated by electrolyte leakage test measurements [58]. Leaf discs of about 0.5 cm^2^ as well as 2 cm long root and stem samples were cut with a razor blade and inserted into a test tube containing 8 mL of distilled water. Samples were stirred for 30 min at room temperature; then, the initial electrical conductivity of the solution (EC_i_) was recorded by a conductivity meter (Cond 5, XS instruments, Carpi, Italy). Samples were then subjected to three freeze-thaw cycles (−20 °C, +20 °C) to induce complete membrane disruption and processed as above to measure the final electrical conductivity of the solution (EC_f_). The relative electrolyte leakage (REL) was calculated as: (EC_i_/EC_f_) × 100.

### 4.4. Structural Traits and Biomass Allocation

Watered samples used for estimating RWC values were also measured for estimating leaf dry matter content, (LDMC) as well as stem (SDMC) and the root (RDMC) dry matter content (as analogue of LDMC). LDMC, SDMC and RDM values were estimated by the ratio between leaf, stem or root dry weight and the corresponding turgid weight. Moreover, stem and leaf dry weight ratio and root and shoot dry weight ratio were also calculated.

Root, stem and leaves dry biomass was estimated by oven drying samples for 3 d at 70 °C.

### 4.5. Plant Hydraulic Conductance Measurements by EFM

Plant hydraulic conductance (K_plant_) values were measured in planta by the Evaporative Flux Method, EFM (35) as:

K_plant_: E_L_/(Ψ_soil_ − Ψ_L_)

where Ψ_soil_ is the soil water potential estimated by a psycrometer (WP4, Decagon Devices, Pullman, WA, USA). All hydraulic conductance values were corrected to a temperature of 20 °C to consider changes in water viscosity.

Hydraulic measurements were estimated in at least 6 samples per species (i.e., *S. ceratophylloides* and *S. officinalis*) and per treatment.

The EFM is expected for providing relative values of hydraulic conductance due to its intrinsic limit in estimating the transpiration of the whole plant. Nevertheless, different studies have reported comparable data between EFM and other hydraulic measurements [59,60,61]. Moreover, the method is considered suitable for comparing values when recorded in similar environmental conditions. Thus, in order to perform reliable hydraulic measurements, the water stress treatment was not imposed the same day in all samples. This experimental procedure allowed us to perform measurements on the same day (and then similar environmental condition) on at least 3 W, 3 S and 3 Rec samples per species and treatment, thus avoiding, as possible, differences in transpiration rate values induced by different boundary layer resistance. Nevertheless, temperature as well as RH values (and then VPD) were similar during the entire experimental period.

### 4.6. Statistical Analysis

Data were analyzed with the SigmaStat 12.0 (SPSS, Inc., Chicago, IL, USA) statistics package. To test leaf structural traits, plant biomass and water storage traits, a *t* test was performed. To test the differences among species (S, i.e., *Sc* and *So*) and the effects of the irrigation treatment (T, i.e., well-watered, W, water-stressed, S and re-irrigated, Rec, samples) on g_L_, E_L_, A_n_, WUE, Ψ_L_, K_plant_, RWC, PLRC and REL a two-way ANOVA test was performed. When the difference was significant, a post hoc Holm–Sidak test was conducted. The significance of correlations was tested using the Pearson product-moment coefficient. Significance was evaluated in all cases at *p* < 0.05. Relationships between K_plant_ and leaf, stem, and root RWC, PLRC and REL values and associate 95% confidence intervals (C.I.s) were assessed in order to obtain species-specific and plant organ specific thresholds. Specifically, values of RWC (RWC_50K_ and RWC_80K_), PLRC (PLRC_50K_, PLRC_80K_) and REL (REL_50K_ and REL_80K_) corresponding to 50% and 80% loss of K_plant_ were estimated for each plant organ in *Sc* and *So*.

## 5. Conclusions

Results recorded in the present study highlight as root plays a key role in plant drought resilience in the two measured *Salvia* species. Large biomass allocation into the root system likely allows to higher accumulation of reserves for sustaining post-drought recovery. Further studies monitoring leaf, stem and root water content and loss in cell rehydration capability can provide important insights, enabling a comprehensive understanding of drought resistance strategies of Mediterranean species, and more accurate prediction of their fate in response to global warming.

## Figures and Tables

**Table 1:** Mean values ± SD of structural and biomass data and water storage traits as measured in well-watered samples of *S. ceratophylloides* and *S. officinalis* plants (*n* = 10). Differences between species were statistically analyzed and corresponding *p* values are reported.

Parameters	*S. ceratophylloides*	*S. officinalis*	*p* Value
	Structural and biomass data		
N leaves/plant	47.7 ± 7.7	164.7 ± 59.3	**<0.001**
A_L_ (cm^2^)	776 ± 80	2788 ± 976	**<0.001**
LDMC	0.16 ± 0.01	0.25 ± 0.03	**<0.001**
DW leaves, g	2.2 ± 0.9	12.7 ± 1.9	**<0.001**
N shoots/plant	2.4 ± 0.5	2.5 ± 1.3	0.411
DW stem, g	0.3 ± 0.1	1.1 ± 0.2	**<0.001**
SDMC	0.22 ± 0.03	0.31 ± 0.03	**<0.001**
Stem/leaf ratio	1.78 ± 0.95	0.95 ± 0.02	**0.007**
DW root, g	1.1 ± 0.4	1.4 ± 0.4	0.104
RDMC	0.14 ± 0.03	0.18 ± 0.01	**0.004**
Root/shoot ratio	2.2 ± 0.9	0.6 ± 0.3	**<0.001**
	Water storage traits		
SWC_leaf_, g g^−1^	4.8 ± 0.4	2.7 ± 0.3	**<0.001**
SWC_stem_, g g^−1^	3.3 ± 0.6	2.4 ± 0.6	**<0.001**
SWC_root_, g g^−1^	5.2 ± 1.4	4.4 ± 0.5	0.061

N leaves/plant: number of leaves per plant; A_L_: whole plant leaf area; LDMC: leaf dry mass content; DW leaves: leaves dry weight per plant; N shoots/plant: number of shoots per plant; DW stem: stems dry weight per plant; SDMC and RDMC: stem and root dry matter content, respectively; DW root: root dry weight; Stem/leaf ratio and Root/shoot ratio: stem/leaf dry weight ratio and root/shoot dry weight ratio; SWC_leaf_, SWC_stem_ and SWC_root_ leaf: stem and root saturated water content, respectively.

## Data Availability

Not applicable.

## Supplementary Materials

The following are available online at https://www.mdpi.com/article/10.3390/plants10091888/s1, Figure S1: g_L_ and Ψ_L_ relationships in *Sc* versus *So* samples; Table S1: RWC, PLRC and REL thresholds of drought-driven K_plant_ decline of *Sc* and *So*.
