# Supplementary material for: When Water Availability Is Low, Two Mediterranean Salvia Species Rely on Root Hydraulics
Source: Plants (Basel). 2021 Sep 13;10(9):1888. doi: 10.3390/plants10091888 (PMC8472023; doi:10.3390/plants10091888)
Supplement: Supplementary file 1 [file plants-10-01888-s001.zip › plants-1339380-supplementary.pdf]

Supplementary Materials

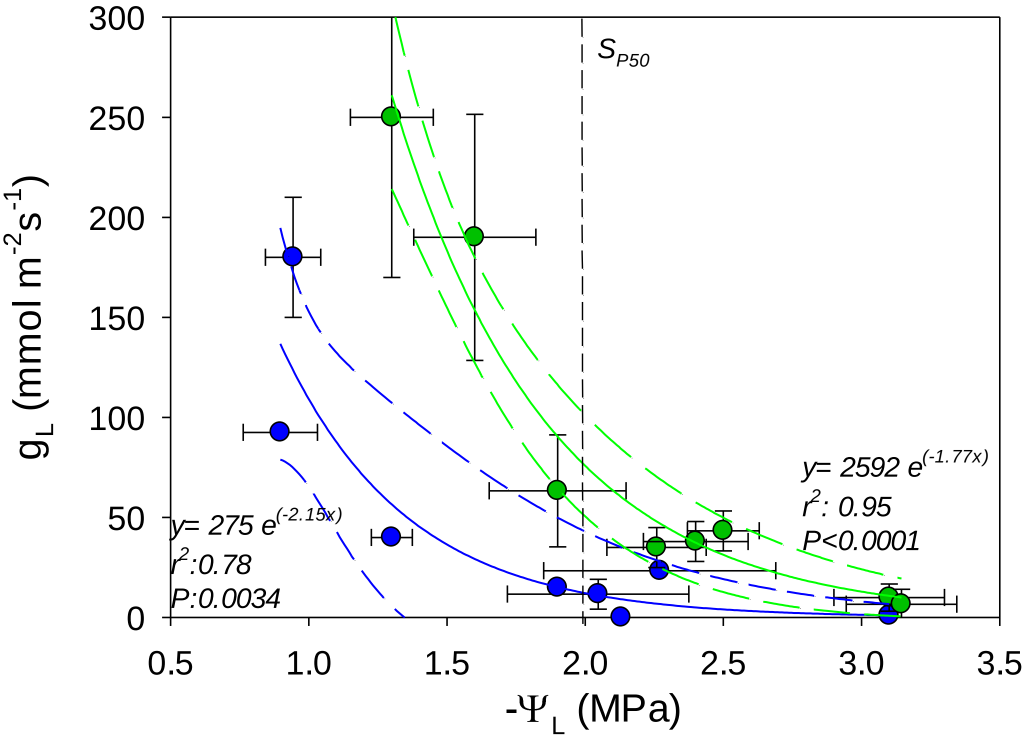

Figure S1: Relationships between mean ± SD values of midday leaf conductance to water vapour (g_L) and leaf water potential (Ψ_L) as recorded in *S. ceratophyllodes* (blue circle symbols) and *S. officinalis* (green circle symbols) samples during dehydration. Solid and long-dashed lines indicate the best-fit regression curve and corresponding 95% confidence intervals, respectively. Short-dashed line indicate the leaf water potential leading to 50% loss of leaf hydraulic conductance (S_{P50}). Regression equation, correlation coefficient (r^2) and P-values are reported.

Table S1: RWC, PLRC and REL values inducing 50% (i.e., RWC_{50K}, $PLRC_{50K}$, REL_{50K}, respectively) and 80% (i.e, RWC_{80K}, $PLRC_{80K}$ and REL_{80K}, respectively) loss of the plant hydraulic conductance as recorded in *S. ceratophylloides* (Sc) and *S. officinalis* (So) plants. Confidence intervals (CI) are also reported.

	**Leaf**				**Stem**				**Root**		
	**RWC_{50K}**	**2.5% CI**	**97.5% CI**		**RWC_{50K}**	**2.5% CI**	**97.5% CI**		**RWC_{50K}**	**2.5% CI**	**97.5% CI**
Sc	64	75	54		67,5	74	62		64	54	69
So	67,5	71	64		62	65,5	58		60	65	52,5
	**RWC_{80K}**	**2.5% CI**	**97.5% CI**		**RWC_{80K}**	**2.5% CI**	**97.5% CI**		**RWC_{80K}**	**2.5% CI**	**97.5% CI**
Sc	35	48	16		44	55	40		38	44	31,5
So	56	61	51		50	55	42,5		31	40	21
	**$PLRC_{50K}$**	**2.5% CI**	**97.5% CI**		**$PLRC_{50K}$**	**2.5% CI**	**97.5% CI**		**$PLRC_{50K}$**	**2.5% CI**	**97.5% CI**
Sc	14	0	50		15	10	21		15	9	25
So	6	4,5	14		14	9	17,5		15	9	24
	**$PLRC_{80K}$**	**2.5% CI**	**97.5% CI**		**$PLRC_{80K}$**	**2.5% CI**	**97.5% CI**		**$PLRC_{80K}$**	**2.5% CI**	**97.5% CI**
Sc	50	14	n.d.		34	21	n.d.		39	25	56
So	19	9	n.d.		24	18	34		30	20	n.d.
	**REL_{50K}**	**2.5% CI**	**97.5% CI**		**REL_{50K}**	**2.5% CI**	**97.5% CI**		**REL_{50K}**	**2.5% CI**	**97.5% CI**
Sc	16	13	25		25,5	20,5	31		30	26	37,5
So	21	15,5	30		26	21	32,5		32,5	29	39
	**REL_{80K}**	**2.5% CI**	**97.5% CI**		**REL_{80K}**	**2.5% CI**	**97.5% CI**		**REL_{80K}**	**2.5% CI**	**97.5% CI**
Sc	31	20	n.d.		38	32	n.d.		51	41	69
So	35	24	n.d.		39	32,5	50		55	45	70
